# *De novo* Transcriptome Assembly and Comparison of C_3_, C_3_-C_4_, and C_4_ Species of Tribe Salsoleae (Chenopodiaceae)

**DOI:** 10.3389/fpls.2017.01939

**Published:** 2017-11-14

**Authors:** Maximilian Lauterbach, Hanno Schmidt, Kumari Billakurthi, Thomas Hankeln, Peter Westhoff, Udo Gowik, Gudrun Kadereit

**Affiliations:** ^1^Institute for Molecular Physiology, Johannes Gutenberg-University Mainz, Mainz, Germany; ^2^Institute for Organismic and Molecular Evolutionary Biology, Johannes Gutenberg-University Mainz, Mainz, Germany; ^3^Senckenberg Biodiversity and Climate Research Centre (SBiK-F), Frankfurt am Main, Germany; ^4^Institute for Developmental and Molecular Biology of Plants, Heinrich-Heine-University Düsseldorf, Düsseldorf, Germany; ^5^Cluster of Excellence on Plant Sciences, Düsseldorf, Germany; ^6^Institute for Biology and Environmental Science (IBU), Plant Evolutionary Genetics, Carl von Ossietzky University Oldenburg, Oldenburg, Germany

**Keywords:** Caryophyllales, evolution, leaf, photorespiration, photosynthesis, RNA-Seq, *Salsola*

## Abstract

C_4_ photosynthesis is a carbon-concentrating mechanism that evolved independently more than 60 times in a wide range of angiosperm lineages. Among other alterations, the evolution of C_4_ from ancestral C_3_ photosynthesis requires changes in the expression of a vast number of genes. Differential gene expression analyses between closely related C_3_ and C_4_ species have significantly increased our understanding of C_4_ functioning and evolution. In Chenopodiaceae, a family that is rich in C_4_ origins and photosynthetic types, the anatomy, physiology and phylogeny of C_4_, C_2_, and C_3_ species of Salsoleae has been studied in great detail, which facilitated the choice of six samples of five representative species with different photosynthetic types for transcriptome comparisons. mRNA from assimilating organs of each species was sequenced in triplicates, and sequence reads were *de novo* assembled. These novel genetic resources were then analyzed to provide a better understanding of differential gene expression between C_3_, C_2_ and C_4_ species. All three analyzed C_4_ species belong to the NADP-ME type as most genes encoding core enzymes of this C_4_ cycle are highly expressed. The abundance of photorespiratory transcripts is decreased compared to the C_3_ and C_2_ species. Like in other C_4_ lineages of Caryophyllales, our results suggest that PEPC1 is the C_4_-specific isoform in Salsoleae. Two recently identified transporters from the PHT4 protein family may not only be related to the C_4_ syndrome, but also active in C_2_ photosynthesis in Salsoleae. In the two populations of the C_2_ species *S. divaricata* transcript abundance of several C_4_ genes are slightly increased, however, a C_4_ cycle is not detectable in the carbon isotope values. Most of the core enzymes of photorespiration are highly increased in the C_2_ species compared to both C_3_ and C_4_ species, confirming a successful establishment of the C_2_ photosynthetic pathway. Furthermore, a function of PEP-CK in C_2_ photosynthesis appears likely, since PEP-CK gene expression is not only increased in *S. divaricata* but also in C_2_ species of other groups.

## Introduction

The convergent evolution of complex traits challenges evolutionary biologists since evolutionary stable intermediate steps seem to be required to accomplish the transition to complex phenotypes (Washburn et al., 2016). In plants, one prime example of such a complex trait is C_4_ photosynthesis. C_4_ photosynthesis evolved more than 60 times in various angiosperm lineages including monocots and eudicots (Sage, 2016). These multiple independent origins of C_4_ photosynthesis from the ancestral C_3_ pathway allow investigating the acquisition of the C_4_ syndrome in individual plant groups and, subsequently, to integrate all acquired components from the different plant groups for understanding the whole complexity and variability of C_4_ evolution. Specifically, C_4_ is a carbon-concentrating mechanism that evolved to cope with decreasing atmospheric CO_2_ concentration (Ehleringer et al., 1991), a condition which would otherwise favor photorespiration particularly in subtropical regions (Bauwe et al., 2010). In mesophyll tissue, atmospheric CO_2_ is initially fixed by phospho*enol*pyruvate carboxylase (PEPC), yielding a compound consisting of four carbon atoms, the key and name-giving step of C_4_ (Hatch, 1987). This C_4_ compound is then modified, transported into the bundle sheath tissue and eventually decarboxylated to increase CO_2_ concentration, allowing a high carboxylation:oxygenation ratio of ribulose-1,5-bisphosphate carboxylase/oxygenase (RuBisCO) in the Calvin-Benson cycle, which results in drastically reduced photorespiration (Hatch, 1987).

The current model of C_4_ evolution predicts a gradual establishment of the C_4_ cycle from C_3_ through a limited number of evolutionary steps (Sage et al., 2012; Bräutigam and Gowik, 2016). Here, the formation of a photorespiratory CO_2_ pump—operating via glycine shuttling by restricting the combined glycine decarboxylase complex (GDC) and serine hydroxymethyltransferase (SHMT) reactions to the bundle sheath—is a major landmark (Sage et al., 2012; Bräutigam and Gowik, 2016). Species exhibiting this glycine shuttle are characterized by lower CO_2_ compensation points than C_3_ plants, a leaf anatomy that is intermediate between C_3_ and C_4_ species, and no or low C_4_ cycle activity (Edwards and Ku, 1987; Sage et al., 2012). The C_3_-C_4_ intermediate species of *Flaveria* that mostly rely on the photorespiratory CO_2_ pump and where C_4_ cycle activity is low are called C_2_ species (Sage et al., 2012). Whether C_3_-C_4_ intermediate phenotypes with or without a photorespiratory CO_2_ pump represent true evolutionary intermediate states for the complex C_4_ syndrome is still under debate and subject of recent investigations (Monson et al., 1984; Monson and Moore, 1989; Sage, 2004; Heckmann et al., 2013; Williams et al., 2013; Bräutigam and Gowik, 2016; Schlüter and Weber, 2016; Kadereit et al., 2017). Although rare in comparison to C_3_ and C_4_ species, the extant C_2_ species clearly represent an established photosynthetic pathway by their mere existence, and also by their phylogenetic age (i.e., several million years) in some lineages (Christin et al., 2011; reviewed in Sage et al., 2012).

Evolution of C_4_ or C_2_ from C_3_ photosynthesis requires many changes, including alterations in leaf anatomy, physiology and gene regulation (Gowik and Westhoff, 2011; Langdale, 2011). Analyses of leaf transcriptomes provided broad knowledge of gene expression in C_4_ and C_2_ photosynthesis by comparing closely related C_3_, C_4_ and/or C_2_ species (Bräutigam et al., 2011; Külahoglu et al., 2014; Mallmann et al., 2014; van den Bergh et al., 2014; Ding et al., 2015; Aubry et al., 2016; Schlüter et al., 2016a,b), leading to the identification of many genes and proteins which function in C_4_ or C_2_ photosynthesis. One general trend, for example, seems to be that most of the key genes of photorespiration, while transcriptionally downregulated in C_4_, are highly expressed in C_2_ when compared to C_3_ plants (reviewed in Bräutigam and Gowik, 2016). Besides genes encoding proteins of the C_4_ cycle and photorespiration, genes related to photosynthesis strongly differ between C_3_ and C_4_ species, at least in the genera *Cleome sensu lato* and *Flaveria* (Bräutigam et al., 2011; Gowik et al., 2011; Aubry et al., 2014; Külahoglu et al., 2014; Kümpers et al., 2017). Gene expression analyses also revealed that C_4_ genes were mostly recruited from expression domains with housekeeping functions (Külahoglu et al., 2014). Additionally, known and novel transporters could be identified, and transport processes in general seem to be very important in C_4_ photosynthesis (Schlüter et al., 2016b).

The goosefoot family (Chenopodiaceae) is an outstanding system to study C_4_ photosynthesis, because it comprises the largest number of both C_4_ species and independent C_4_ origins in the eudicots, with an outstanding diversity of the C_4_ phenotype (Kadereit et al., 2003, 2010, 2014; Kadereit and Freitag, 2011; Sage, 2016). Additionally, Chenopodiaceae contain a number of unique study systems, e.g., single cell C_4_ plants, like the genus *Bienertia* (Freitag and Stichler, 2002) or *Suaeda aralocaspica* (Freitag and Stichler, 2000), the stem succulent C_4_ hygro-halophytes *Tecticornia indica* and *T. bibenda* (Shepherd and van Leeuwen, 2007; Voznesenskaya et al., 2008) and the species of tribe Salsoleae that conduct C_3_ in cotyledons before they switch to C_4_ in leaves or assimilating shoots (Voznesenskaya et al., 2013; Li et al., 2015; Lauterbach et al., 2016). Furthermore, Salsoleae seem particularly suitable to study the evolution of C_2_ and C_4_ photosynthesis because the tribe contains a comparatively large number of C_2_ species (summarized in Voznesenskaya et al., 2013 and Schüssler et al., 2016). Salsoleae are widespread in semi-deserts, deserts and coastal regions of Eurasia and well-adapted to dry and saline conditions (Akhani et al., 2007). Their leaves and/or shoots show a central water storage tissue. Often the leaves are reduced and photosynthesis is taken over by the shoots (Schüssler et al., 2016). Leaves or assimilating shoots often have a multi-layered epidermis and a hypodermis. The chlorenchyma forms a continuous layer surrounding the entire leaf and consists of 2-3 mesophyll layers in C_3_ species and one outer mesophyll layer and a Kranz layer in C_4_ species (Salsoloid leaf anatomy), respectively (Carolin et al., 1975). In the C_3_-C_4_ intermediate species in general, the Kranz layer is either continuous or interrupted by water storage cells, and the mesophyll can consist of two layers (Voznesenskaya et al., 2013; Schüssler et al., 2016). Recently, a molecular phylogeny including the major lineages of Salsoleae has been published (Schüssler et al., 2016) which is the basis for the phylogenetically informed sampling of this study. Despite the high diversity in this group, large-scale genomic analyses of coding sequence information are currently limited to two taxa, *Haloxylon ammodendron* (Li et al., 2015) and *Salsola soda* (Lauterbach et al., 2016).

Here we present transcriptome *de novo* assemblies and the analyses of differential gene expression of representative species of Salsoleae with different photosynthetic types, including C_3_, C_4_, and C_2_ photosynthesis. In particular, we sequenced the transcriptomes of the main assimilating organs (i.e., leaf or assimilating shoot) of the C_3_ species *Salsola webbii*, two distinct populations of the C_2_ species *S. divaricata*, the C_4_ species *S. oppositifolia*, and *Hammada scoparia*, which conducts C_3_ in cotyledons, but C_4_ in assimilating shoots. Additionally, we included publicly available leaf transcriptome data from *Salsola soda*, which also exhibits C_3_ in cotyledons and C_4_ in leaves (Lauterbach et al., 2016). Transcriptomes of all five species were assembled *de novo* and gene expression patterns between the species/populations were compared with a focus on genes involved in C_4_ photosynthesis and photorespiration to address the question whether gene expression profiles of C_3_, C_2_, and C_4_ species of Salsoleae are comparable to other study systems like *Cleome* or *Flaveria*.

## Materials and methods

### Plant material

**Figure 1:**
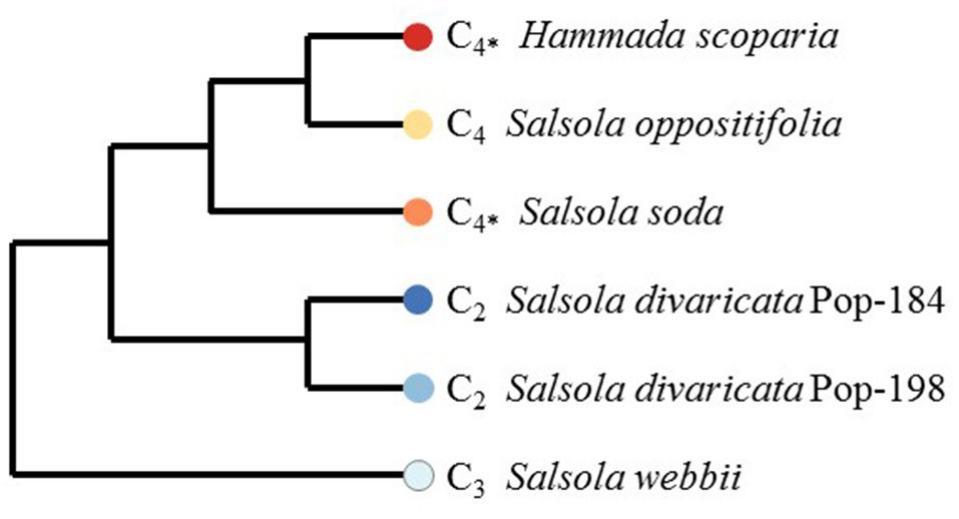
Highly reduced phylogenetic tree including only species of the current study [adapted and modified from Open Access article by Schüssler et al. (2016), which permits unrestricted reuse; C_4*_, species with C_4_ photosynthesis in leaves/assimilation shoots but C_3_ in cotyledons].

Seeds were taken from plants collected in the field, and vouchers of these collections are deposited at the herbarium of Johannes Gutenberg-University Mainz (MJG; see Table 1 for further information and a comment on taxonomic and nomenclatural issues in Salsoleae). Plants of *H. scoparia, Salsola divaricata* (from two different populations: population 184 (Pop-184) located in Lanzarote and population 198 (Pop-198) located in Gran Canaria), *S. oppositifolia*, and *S. webbii* were grown from seeds in potting soil (custom mixed soil from the Botanic Garden, Johannes Gutenberg-University Mainz) in a glasshouse with an additional light intensity of ca. 300 μmol m^−2^ s^−1^. Samples were harvested between 16th April and 16th May 2014 between 10:30 and 13:00, immediately frozen in liquid nitrogen and stored at −80°C for RNA extraction. A highly reduced phylogenetic tree based on the results of Schüssler et al. (2016) including only species of the current study is shown in Figure 1.

**Table 1:** Species of Salsoleae *sensu stricto* included in the study.

Species^*^	**PS type**	**δ^13^C of leaf (*n* = 3)**	**Herbarium and voucher ID**
*Hammada scoparia* (Pomel) Iljin^**^	C_4_	−18.759	MJG living collection no. 87 (source MSB serial no. 89920; Morocco: Taroudannt)
*Salsola divaricata* Moq.; Population184	C_2_	−32.208	MJG Herbarium no. 014225, living collection no. 184 (source: Canary Islands: Lanzarote, Orzóla)
*Salsola divaricata* Moq.; Population198	C_2_	−31.759	MJG living collection no. 198 (source: Canary Islands: Gran Canaria, Cuesta Ramón, Jinamar)
*Salsola oppositifolia* Desf.	C_4_	−17.514	MJG Herbarium no. 013564, living coll. No. 173 (source: SW Morocco, 18 km N Agadir, near Tamrhakt)
*Salsola soda* L.	C_4_	−15.434^***^	MJG no. 014562 (see Lauterbach et al., 2016)
*Salsola webbii* Moq.	C_3_	−31.712	MJG living collection no. 67 (source: G. Edwards lab, Pullman, Washington, originally collected in S. Spain)

* *The highly polyphyletic genus Salsola and also the Salsoleae are currently experiencing dramatic taxonomic and nomenclatural rearrangements (compare Akhani et al., 2007, 2016; Hernández-Ledesma et al., 2015; Mosyakin et al., 2017). For the sake of easy comparability for non-Salsoleae experts we therefore prefer to use the established names in this paper*.

** *sometimes treated as synonym of Hammada articulata O. Bolòs and Vigo*.

*** *measured in Lauterbach et al. (2016)*.

### Carbon isotope discrimination measurements

Leaves or, in case of *H. scoparia*, assimilation shoots of all five species were harvested and dried for several days in silica gel. Dry leaf samples were pulverized using the mixer mill MM 301 (Retsch). Approximately 200 mg of each sample were used to determine stable carbon isotope ratios (i.e., 13C/12C) relative to the Pee Dee belemnite standard (Craig, 1957) by the Institute for Geosciences at Johannes Gutenberg-University Mainz. For each sample, technical triplicates were measured.

### RNA sequencing

RNA extraction, library preparation and mRNA sequencing were performed as described in Lauterbach et al. (2016). In brief, total RNA was extracted from 9 to 90 mg cotyledon or leaf tissue using the RNeasy Plant Mini Kit (Qiagen), including DNase I digestion with RNase-Free DNase Set (Qiagen). After quality control of RNA using the 2100 Bioanalyzer (Agilent Technologies), NanoDrop (Thermo Fisher Scientific) and Qubit (Life Technologies), 500 ng of total RNA were used for cDNA library preparation with the TruSeq RNA Sample Preparation Kit (Illumina Inc.), following the Low Sample Protocol (TruSeq RNA Sample Preparation v2, May 2012). Sequencing of 101 bp single-end reads was performed on an Illumina HiSeq2000 platform.

For RNA-Seq, different tissues were sampled: (1) assimilation shoots of *H. scoparia*, (2) cotyledons and leaves of *S. divaricata* Pop-184, (3) cotyledons and leaves of *S. divaricata* Pop-198, (4) cotyledons and leaves of *S. oppositifolia*, (5) cotyledons and leaves of *S. soda* (RNA-Seq data taken from Lauterbach et al., 2016), (6) leaves of *S. webbii* (see Table 1 for further details). Three biological replicates (i.e., triplicates) per species/population and organ were sequenced, except for *S. webbii* where only two biological replicates and one technical replicate were sequenced due to the lack of a third individual in the living collection at Botanic Garden, Johannes Gutenberg-University Mainz. Additionally, for *H. scoparia* and *S. webbii* no seeds were available, thus only leaves could be sampled. Both cotyledons were sampled for cotyledon samples while a single leaf was sampled for leaf samples.

### Processing of raw reads and *de novo* transcriptome assembly

Sequence reads were quality controlled with the FASTQC tool (http://www.bioinformatics.babraham.ac.uk/projects/fastqc/), and filtered and trimmed using the tools “fastx_clipper” (option -M 15 -l 20) to remove adapter sequences, “fastx_trimmer” (option -f 15) to remove the first 14 bases of the 5' end of all reads, “fastq_quality_trimmer” (option -t 20 -l 20) to remove low quality bases (below PHRED score of 20) from the 3' end, and “fastq_quality_filter” (option -q 20 -p 80) to remove all reads with an overall PHRED score below 20. All tools are from the FASTX toolkit (http://hannonlab.cshl.edu/fastx_toolkit/) and were used in the same order as listed.

Initially, we conducted *de novo* transcriptome assemblies for each species with two different tools, SOAPdenovo-Trans v1.04 (Xie et al., 2014) and Trinity v2.1.1 (Grabherr et al., 2011), since both were reported to perform well in *de novo* assembling transcriptome data (Honaas et al., 2016; Wang and Gribskov, 2016). Default parameters were used, except for kmer size = 73 in SOAPdenovo-Trans-127mer. The qualities of *de novo* transcriptome assemblies were assessed using three different approaches. Firstly, BUSCO v.2.0 (Benchmarking Universal Single-Copy Orthologs; Simão et al., 2015) with the plant early release data set was used as a proxy for the completeness of the assembled transcriptomes. The BUSCO tool evaluates the completeness of the expected gene content with hidden Markov models (using HMMER v3.1; hmmer.org) and lineage-specific BUSCO profiles (Simão et al., 2015). Secondly, we mapped all quality controlled reads back to the corresponding *de novo* transcriptome assembly using bowtie2 v.2.1.0 (Langmead and Salzberg, 2012) for inferring the percentage of back-mapped reads as a measure of quality. Finally, we performed a sequential BLASTx search (cut-off e-value of 1e-10, and keeping only best hit) against coding sequences of the minimal *Arabidopsis thaliana* transcriptome (Bräutigam et al., 2011), the *Beta vulgaris* transcriptome (version “BeetSet-2”, Dohm et al., 2014) and the UniProtKB database (http://www.uniprot.org/) to see how many contigs were most similar to genes with a known function/annotation (i.e., putative genes). Here, all contigs firstly were mapped again Arabidopsis, the remaining unmapped contigs were mapped against *B. vulgaris*, and again remaining unmapped contigs were mapped against UniProtKB. *A. thaliana* was chosen because this species has the best-annotated plant genome, and *B. vulgaris* was chosen because this is the phylogenetically closest species to tribe Salsoleae with a sequenced genome.

After deciding to use the Trinity assemblies (see next paragraph), functional annotation of transcripts was performed via BLAST-based comparison (see above) against the Arabidopsis and *B. vulgaris* transcriptomes and the UniProtKB database. Sets of annotated transcripts served as reference transcriptomes for inferring differential gene expression (see below). Contigs from different species producing the same annotation match in either Arabidopsis, *B. vulgaris* or UniProtKB databases were defined as putative orthologs. As additional assessment for defined putative orthologs, pairwise reciprocal BLAST (tBASTx, cut-off *e*-value of 1e-10) between all six *de novo* assemblies were performed, and read counts especially of genes involved in C_4_ photosynthesis and photorespiration were manually double-checked. To exclude the presence of multiple putative loci per real locus and due to that erroneous read counts, unique presence of genes encoding proteins involved in C_4_ photosynthesis and photorespiration were manually checked. Venn diagrams showing the overlap of the putative orthologous contigs between all assemblies were calculated and drawn using the “venn” package (by Adrian Dusa, University of Bucharest, Romania) in R (R Core Team, 2016).

### Quality assessment of *de novo* transcript assemblies

*De novo* assemblies were conducted with at least 97.8 million (*S. webbii*) and up to 205 million (*S. oppositifolia*) quality-controlled reads. While we only used data from leaf samples for differential gene expression analysis, we included additional reads from cotyledon samples (Lauterbach et al., in preparation) for the *de novo* assemblies of *S. divaricata* Pop-184 and Pop-198, *S. oppositifolia*, and *S. soda* transcriptomes (see above).

Results of both assemblers, SOAPdenovo-Trans and Trinity, yielded partly different results (Table 2, Supplementary Table S1, Supplementary Figure S1). We decided to continue downstream analyses with the Trinity assemblies for several reasons. First, the number of assembled bases was at least twice as high in the Trinity assemblies compared to SOAPdenovo-Trans assemblies. Second, assessment with BUSCO showed that the completeness in terms of expected gene content was considerably higher in the Trinity assemblies (Supplementary Figure S2). Third, the amount of mappable reads was higher in the Trinity assemblies, which is an important metric to identify leading assemblers. Finally, contigs of the Trinity assemblies matched more proteins with known annotation of the three references (Supplementary Figure S3).

**Table 2:** Summary of statistics of *de novo* transcriptome assemblies using SOAPdenovo-Trans and Trinity (BUSCO, Benchmarking Universal Single-Copy Orthologs).

	**No. of assembled contigs**	**Mean length (in bp)**	**No. of assembled bases (in million)**	**Complete BUSCOs (in %)**	**Fragmented BUSCOs (in %)**	**Missing BUSCOs (in %)**	**Back mapping rate (in %)**
SOAPdenovo-Trans	56,756–186,466	333.87–682.05	37–84.4	47.9–87.4	6.3–37.8	6.3–17.5	84.9–96.3
Trinity	127,382–465,856	659.49–951.37	119.1–302	89.6–93.9	2.9–7.0	2.8–4.4	94.1–98.4

### Statistics, data analysis, and differential gene expression

For studying differential gene expression, processed reads were mapped against the respective *de novo* transcriptome reference assemblies using bwa-mem v.0.7.13 (Li and Durbin, 2009). To infer quantitative information about transcript abundance, reads that uniquely mapped to one contig (i.e., excluding unmapped reads and multi-mapped reads) were extracted by Samtools v.1.3 (Li and Durbin, 2009). These counts were further used for comparison between replicates and species. RNA-Seq data of the six leaf samples were comparatively analyzed to detect differential gene expression. Pairwise comparisons between all six samples were statistically evaluated using “edgeR” (Robinson et al., 2010) in R. After normalization using the *trimmed mean of M-values* (TMM) method in edgeR, only contigs that had at least five reads per million in at least three samples (including replicates) were considered. Log_2_ transcript ratios were calculated and a log_2_ fold change (log_2_FC) of ≥1 was applied as threshold to call differentially expressed genes. A significance threshold of 0.01 was defined after Benjamini-Hochberg correction to account for multiple testing (Benjamini and Hochberg, 1995) and applied to call differentially expressed genes. To further analyze and visualize the data, log_2_ transformed read counts (transcripts per million) were used for hierarchical clustering using Pearson correlation and average linking method (MultiExperiment Viewer, MeV v.4.9, http://mev.tm4.org/) and principal component analysis (PCA, MeV). Additionally, hierarchical clusterings of transcript abundance of selected genes encoding known C_4_ cycle proteins (Lauterbach et al., 2016) and the eight core enzymes of photorespiration (Bauwe et al., 2010) were conducted (Pearson correlation, average linkage method; MeV).

## Results

### Carbon isotope discrimination

Carbon isotope values varied between −15.434 and −32.208 (Table 1). Leaves of *S. divaricata* Pop-184, *S. divaricata* Pop-198, and *S. webbii* had values of −31.712, −32.208, and −31.759, respectively, indicating the absence of a high-activity C_4_ cycle, while leaves of *H. scoparia, S. oppositifolia* and *S. soda* had values of −18.759, −17.514, and −15.434, respectively, indicating a predominating C_4_ metabolism (Table 1). The values for *S. oppositifolia* and *H. scoparia* appear a bit too negative, maybe this is due to the fact that these were relatively young plants raised in the greenhouse. According to values in the literature (Schüssler et al., 2016, and references therein) both species are clearly C_4_ plants.

### RNA sequencing

In total, RNA sequencing yielded between 29.6 and 39.4 million single-end raw reads per replicate, with at least 97.8% reads remaining after quality filtering (Supplementary Table S1, Supplementary Figure S4). Raw data is available in the ArrayExpress database at EMBL-EBI (www.ebi.ac.uk/arrayexpress) under accession number E-MTAB-5778.

### Differences in gene expression between the species

After deciding to use the annotated Trinity assemblies as reference transcriptomes, quality-filtered reads of the different RNA-Seq samples were mapped and counted using bwa-mem. 48,818 contigs had a reliable annotation in one of the six samples; of these, 15,064 contigs have an Arabidopsis annotation, 19,108 contigs have a *B. vulgaris* annotation, and 14,646 contigs have a UniProtKB annotation. Of the 48,818 contigs with a reliable annotation in one of the six samples (i.e., putative genes), 34,022 had at least five reads per million in at least three samples, and downstream analyses were limited to this subset.

**Figure 2:**
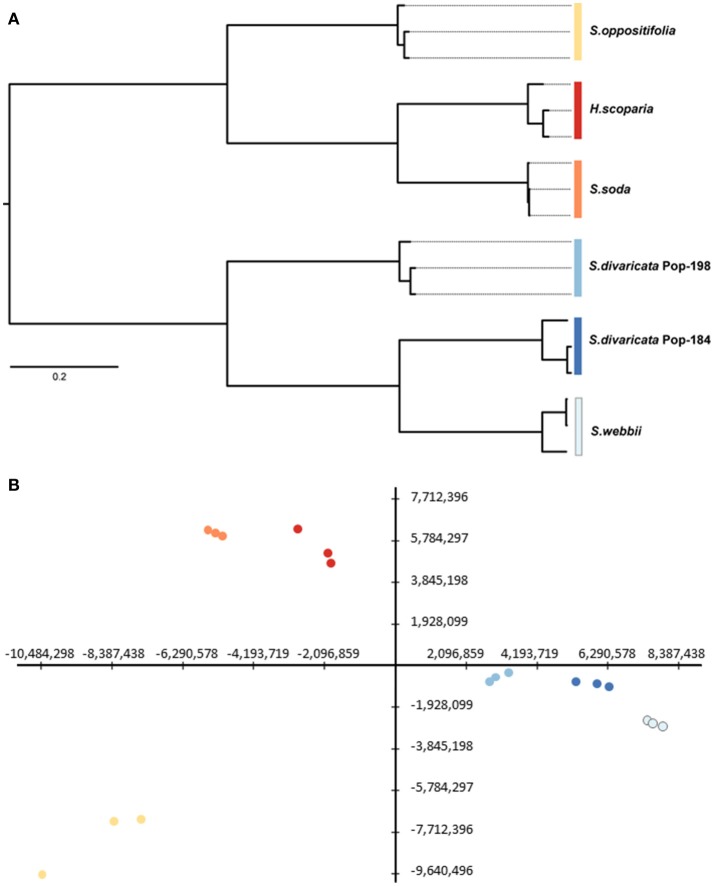
**(A)** Hierarchical clustering using Pearson correlation and **(B)** principal component analysis showing the first (X-axis) and second (Y-axis) components, which explain 57.41% of the total variation (see Supplementary Figure S5 for the first three components), of gene expression data. (The colors in **B**) are the same as in **(A)** and corresponds to replicates of the respective species).

According to their transcript profiles, replicates of each sample clearly grouped together in hierarchical clustering (Figure 2A). The trend in the hierarchical clustering also mostly reflected the phylogenetic relatedness of samples (Figure 1). Thus, the two different populations of the C_2_ species *S. divaricata* grouped together, a result that reflects both phylogenetic relationship and photosynthetic type. The same was observed for the three C_4_ species *H. scoparia, S. oppositifolia*, and *S. soda*, which grouped together (Figures 1, 2A). *H. scoparia* and *S. soda* share a similar photosynthetic phenotype (i.e., C_4_ in leaves/assimilation shoots but C_3_ in cotyledons), and based on their transcript expression profiles they group together (Figure 2A).

Results of the PCA in general supported the results of hierarchical clustering (Figure 2B). All replicates of the same species grouped closely together. The first two components explained 60.58% (Figure 2B), and the first three components explained 76.32% (Supplementary Figure S5) of the total variance (principal component 1: 36.56%; principal component 2: 24.01%; principal component 3: 15.74%; Figure 2B, Supplementary Figure S5). The samples of *H. scoparia* and *S. soda* showed close similarity. The two populations of *S. divaricata* grouped together in hierarchical clustering, results of PCA showed *S. divaricata* Pop-184 as somewhat closer to the C_3_ species *S. webbii* than the Pop-198 sample to *S. webbii*. As in the hierarchical clustering, *S. oppositifolia* was clearly distinct from the other two C_4_ species and also from both populations of *S. divaricata* and from *S. webbii*. The first component of the PCA separated the species based on the photosynthetic type (the three C_4_ species on the left side, C_3_ species on the right, and the two samples of the C_3_-C_4_ intermediate species in between) (Figure 2).

### Transcript abundance among photosynthetic types

Differentially expressed transcripts, defined by adjusted *P*-value ≤ 0.01 and log_2_FC ≥1, were identified by pairwise comparisons between all samples. By comparing differentially expressed transcripts between *S. webbii* (C_3_) and all three C_4_ species *H. scoparia, S. oppositifolia* and *S. soda*, 4,436 genes were found significantly up-regulated in *S. webbii* while 1,907 genes were up-regulated in all three C_4_ species (Supplementary Table S2). Comparing significantly differentially expressed transcripts between C_3_ (*S. webbii*) and C_2_ photosynthesis (*S. divaricata* populations), 6,304 genes were up- and 5,320 genes down-regulated in *S. webbii* (Supplementary Table S2). 1,714 genes were significantly up- and 856 down-regulated in C_2_ *S. divaricata* when compared with all three C_4_ species (Supplementary Table S2).

### Transcript abundance of C_4_-related genes

**Figure 3:**
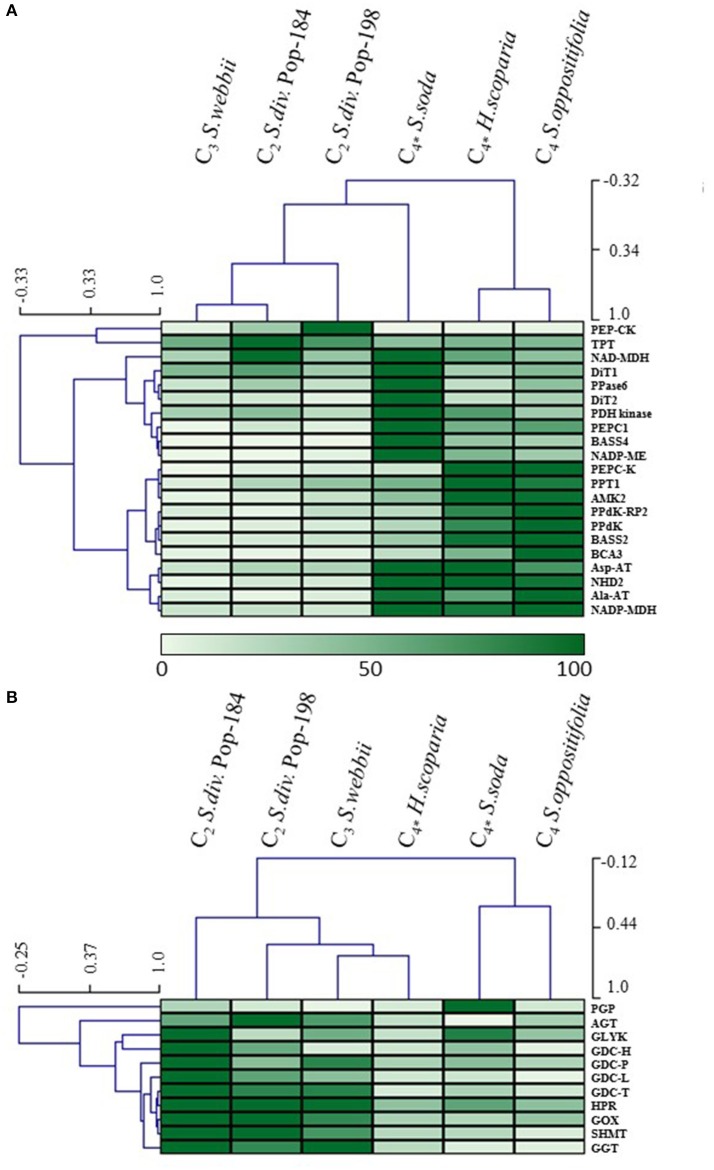
Hierarchical clustering and heatmap of relative transcript abundance of genes encoding **(A)** known C_4_ cycle proteins and **(B)** the eight core enzymes of photorespiration (C_4*_, species with C_4_ photosynthesis in leaves/assimilation shoots but C_3_ in cotyledons; *S. div, S. divaricata*; for description of abbreviated genes see text).

Transcript abundances of 21 genes encoding known C_4_ cycle proteins were analyzed between the species with focus on the different photosynthetic types (Figure 3A). As expected, transcripts encoding the C_4_ cycle proteins alanine aminotransferase 1 (Ala-AT1), adenosine monophosphate kinase 2 (AMK2), aspartate aminotransferase 5 (Asp-AT5), bile acid:sodium symporter family protein (BASS) 2, BASS4, beta carbonic anhydrase 3 (BCA3), NADP-dependent malate dehydrogenase (NADP-MDH), NADP-malic enzyme (NADP-ME), PEPC1, PEPC kinase (PEPC-K), pyruvate orthophosphate dikinase (PPdK), PEP/phosphate translocator (PPT) 1, and sodium:hydrogen antiporter 2 (NHD2) were significantly (FDR *q*–values ≤ 0.01) more abundant in all three C_4_ species compared to C_3_ *S. webbii* (log_2_FC between 1.26 and 7.33; Supplementary Table S2). Increased abundance of transcripts encoding AMK2, BASS4, NHD2, PEPC1, PEPC-K, and PPT1 was found in the three C_4_ species and both populations of *S. divaricata* (C_2_) as compared to *S. webbii* (C_3_). No known C_4_ cycle gene was significantly up-regulated in the C_3_ species *S. webbii* when compared to all other samples.

In addition to the genes that were increased in *all three* C_4_ species when compared to C_3_ *S. webbii* (see above), several C_4_ related genes were increased in one or two of the three C_4_ species only: Transcripts of dicarboxylate transporter (DiT) 2 and pyrophosphatase 6 (PPase6) were more abundant in *S. oppositifolia* and *S. soda* (log_2_FC between 1.08 and 2.35), and transcripts of PPdK regulatory protein (PPdK-RP) were more abundant in *H. scoparia* and *S. oppositifolia* (log_2_FC ≥2.41; Supplementary Table S2). Further, transcripts encoding NAD-dependent malate dehydrogenase (NAD-MDH) were more abundant in *S. soda* (log_2_FC ≥1.41). As expected in C_4_ species, many of the genes encoding C_4_ cycle proteins were among the most highly expressed transcripts in the three C_4_ species. For example, PPdK was the most highly expressed gene in *S. oppositifolia* and the second most highly expressed gene in *H. scoparia*. Other highly expressed genes in the three C_4_ species were Ala-AT, Asp-AT, NADP-ME, NADP-MDH, and PEPC. The NADP-ME subtype of C_4_ photosynthesis is known from C_4_ species of the Salsoleae (Akhani et al., 2007). In accordance, we found high abundance of transcripts encoding NADP-ME in the three C_4_ species, while those of NAD-malic enzyme (NAD-ME) and PEP-carboxykinase (PEP-CK) were low.

In the C_2_ species *S. dicaricata*, AMK2, BASS4, NHD2, PEPC1, PEPC-K, and PPT1 were significantly more abundant compared to *S. webbii* (log_2_FC between 1.12 and 4.43). Interestingly, PEP-CK was more abundant in *S. divaricata* compared to all other species (log_2_FC between 2.72 and 7.26). Additionally, in *S. divaricata* Pop-184, transcripts of NAD-MDH, PPase6, and triose phosphate transclocator (TPT) were significantly up-regulated when compared to *S. webbii* (log_2_FC ≥1.1), while NADP-ME and PPdK were up-regulated in *S. divaricata* Pop-198 in comparison to *S. webbii* (log_2_FC ≥1.5) (Supplementary Table S2). The gene encoding the transporter protein TPT, which is known to function in the C_4_ cycle, was highly abundant in all six samples (between 51st and 82nd highest expressed gene).

In addition to the 21 genes known to be involved in C_4_ photosynthesis (see above), transcripts of genes currently described to have an alleged function related to C_4_ metabolism (Lauterbach et al., 2016) were analyzed. Transcripts encoding pyruvate dehydrogenase (PDH) kinase, probably controlling metabolite exit from the C_4_ pathway via pyruvate decarboxylation (Bräutigam et al., 2014), were significantly more abundant in *S. soda* (log_2_FC 1.29) when compared to *S. webbii*, but still abundant in all other species, including C_3_ *S. webbii*. Phosphate transporter4.1 (PHT4.1) was significantly more abundant in all species (in *S. soda* with log_2_FC of 0.8) compared to *S. webbii*. The gene encoding PHT4.4 was highly upregulated in all three C_4_ species compared to *S. webbii* (log_2_FC ≥1.85). Levels of mRNA coding for asparagine synthetase (ASN) were increased in *S. oppositifolia* and *S. soda* when compared to *S. webbii* (log_2_FC 1.45 and 5.89, respectively). Also, ASN was the 14th highest expressed gene in *S. soda* (Supplementary Table S2).

### Transcript abundance of genes encoding key enzymes of photorespiration

Since photorespiration has been reported as strongly modified between closely related C_3_ and C_4_ species (e.g., *Cleome*, Bräutigam et al., 2011) and also between C_3_, C_2_, and C_4_ species (e.g., *Flaveria*; Gowik et al., 2011; Mallmann et al., 2014), the transcript abundance of the eight core enzymes of photorespiration was analyzed and compared between species (Figure 3B). Transcripts encoding phosphoglycolate phosphatase (PGP) were significantly more abundant in all three C_4_ species compared to *S. webbii* (log_2_FC between 1.16 and 4.73), and gene expression of seven genes were increased in *S. webbii* in comparison to all three C_4_ species: serine:glyoxylate aminotransferase (AGT/SGT), GDC P-protein (GDC-P), GDC L-protein (GDC-L), GDC T-protein (GDC-T), glutamate:glyoxylate aminotransferase (GGT), NADP-dependent hydroxypyruvate reductase (HPR), and SHMT (Supplementary Table S2). The two genes encoding GDC H-protein (GDC-H) and PGP each were significantly up-regulated in *S. divaricata* compared to *S. webbii* (log_2_FC ≥1.96), the former also significantly up-regulated when compared to the three C_4_ species (log_2_FC ≥1.01). Further, *S. divaricata* Pop-184 showed an increase of transcripts encoding GDC-H, glycerate 3-kinase (GLYK) and PGP when compared to *S. divaricata* Pop-198 and *S. webbii* (Supplementary Table S2). In general, all eight genes encoding key enzymes of photorespiration were highly expressed in *S. divaricata* (Pop-184 and 198) and *S. webbii*. Interestingly, transcripts of GDC-P were not only highly abundant in the C_2_ and C_3_ species, but in all six samples.

## Discussion

Both C_2_ and C_4_ photosynthesis are complex and require many changes in gene expression patterns compared to the ancestral C_3_ photosynthesis (Sage, 2004). RNA-Seq strongly promoted the understanding of C_2_ and C_4_ evolution in the last few years. In eudicots, the flagship group to decipher differential gene expression leading to C_2_ or C_4_ photosynthesis is *Flaveria* (Asteraceae), which includes, besides C_3_ species, many C_2_ and C_4_ species (Edwards and Ku, 1987). Likewise, tribe Salsoleae (Chenopodiaceae) comprises C_3_, some C_2_ and an immense diversity of C_4_ lineages (Schüssler et al., 2016). However, it differs significantly in leaf anatomy and ecology from the model genus *Flaveria*, making Salsoleae a promising candidate for increasing our knowledge of convergent acquisition of C_2_ and C_4_ photosynthesis.

### A novel transcriptome data resource for salsoleae

Until now, no transcriptome assembly of tribe Salsoleae (Chenopodiaceae) has been available and large-scale gene expression studies based on RNA sequencing were for the most part absent in this group (Li et al., 2015; Lauterbach et al., 2016). Thus, we used a *de novo* assembly approach to produce reference transcriptomes of six samples representing five species of Salsoleae, thereby providing a novel and large mRNA data resource for this group. Initially applying two different tools, the Trinity assembler was selected because of superior results (see Results; Table 2). *De novo* assemblies of the two different populations of the same species, *S. divaricata*, yielded about the same amount of contigs, implying a consistent performance of Trinity. The number of cDNA contigs obtained did not correlate with the diversity of tissues included in the sequencing: While only stem or leaf tissue of adult plants was available for *H. scoparia* and *S. webbii*, respectively, the other four assemblies, which included cotyledon and adult leaf tissues, yielded fewer (*S. soda*) or more contigs (*S. divaricata* Pop-184, *S. divaricata* Pop-198, *S. oppositifolia*). Interestingly, we obtained more than twice as many contigs from *S. oppositifolia* compared to *S. soda*. This huge difference could potentially have been caused by different ploidy levels in these species: *Salsola oppositifolia* is octoploid (Lago and Castroviejo, 1992; Peruzzi and Cesca, 2004), while *S. divaricata* and *S. webbii* are tetraploid (G. Kadereit and D. Tefarikis; pers. observation; Padrón, 2012) and *S. soda* and *H. scoparia* are diploid (Löve, 1970, 1986; Tarnavschi and Lungeanu, 1982; Zakharyeva, 1985).

Physiological studies (Voznesenskaya et al., 2013) identified *S. webbii* as a typical C_3_ plant, which is why this species was selected as a C_3_ reference and gene expression in leaves of *S. webbii* was taken as exemplary for C_3_ in all downstream analyses. The transcriptome data of *S. webbii* differed strongly from all other species included (Figure 2), which reflects both phylogenetic position and photosynthetic type (Figure 1). Additionally, stable carbon isotope values of leaves (or in case of *H. scoparia* assimilation shoots) were measured since stable carbon isotopes can be used as an indicator of C_4_ metabolism (O'Leary, 1981; Cernusak et al., 2013). All measurements of the species of the current study were consistent with published data (Akhani et al., 1997; Pyankov et al., 2001; Voznesenskaya et al., 2013; Schüssler et al., 2016): *H. scoparia, S. oppositifolia*, and *S. soda* showed C_4_ values, while *S. webbii* and both populations of *S. divaricata* had C_3_-like stable carbon isotope values. Based on anatomical findings and gas exchange measurements, *S. divaricata* was previously shown to conduct C_2_ photosynthesis (Voznesenskaya et al., 2013), which is hardly detectable in stable carbon isotope measurements.

### Gene expression broadly reflects photosynthesis type in salsoleae

Intriguingly, the first principal component of a PCA based on the gene expression data separated the species based on photosynthesis types, with both C_2_ *S. divaricata* samples lying in between the three C_4_ species and C_3_ *S. webbii*. Thus, other variables, for instance different life cycles (*S. soda*: annual vs. all other included species: perennial) or sampled organ (*H. scoparia*: stem vs. leaf in all others), seemed to be of minor importance for differences in gene expression, whereas photosynthesis type was the dominant factor. By comparison, in a PCA of gene expression data of several closely related species of *Flaveria* with different photosynthesis types, the first three components explained only 27% of the total variance (Mallmann et al., 2014). In contrast, the first three components in our own PCA explained about 73% of the total variance. On the one hand this difference could imply a lower gene regulatory diversity among Salsoleae species than among species of *Flaveria*. However, the older evolutionary age of Salsoleae would argue against this assumption. Another explanation might be that other confounding factors influenced gene expression in the *Flaveria* study, while in Salsoleae the different photosynthesis types largely shaped the major gene expression pattern. This is even true for the C_4_ taxa *H. scoparia, S. oppositifolia*, and *S. soda*, which differ from each other morphologically and physiologically: e.g., *H. scoparia* has completely reduced leaves and photosynthesis is taken over by the shoots, and *S. soda* has an annual life cycle while the other two are perennial. Also, *H. scoparia* and *S. soda* convert from C_3_ during cotyledon stage to C_4_ in mature stage (Lauterbach et al., 2016). In terms of C_4_ photosynthesis, however, the three species are alike in most characters. All have Salsoloid leaf (or stem) anatomy, which is the typical C_4_ anatomy in this group (Voznesenskaya et al., 2013; Lauterbach et al., 2016; Schüssler et al., 2016). Also, all belong to the NADP-ME subtype (Akhani et al., 2007; current study). Gene expression levels of most C_4_ genes were thus highly abundant, and at the same time expression of most of the major photorespiratory enzymes was significantly decreased in all three C_4_ species compared to C_3_ *S. webbii*. This important result is in full agreement with studies comparing closely related C_3_ and C_4_ species in spiderflowers (*Cleome sensu lato*, Bräutigam et al., 2011; Aubry et al., 2014; Külahoglu et al., 2014) and *Flaveria* (Gowik et al., 2011).

### Transcriptome analysis reveals specific features of the photosynthesis phenotypes in salsoleae

One key enzyme of C_4_, PEPC, is known to be present in the plant genome in multiple copies, and while different isoforms function in different tissues, only one is recruited into the C_4_ cycle (Westhoff and Gowik, 2004). Here, we provide evidence that PEPC1 is the C_4_-specific isoform in Salsoleae. PEPC1 is one of the top-expressed genes in *H. scoparia, S. oppositifolia*, and *S. soda* and about 25-times less expressed in *S. webbii*, which is in accordance with high PEPC expression in other phylogenetically distant C_4_ species like *Flaveria bidentis* (Gowik et al., 2011). In agreement with these findings, leaves of *S. oppositifolia* were recently shown to have high levels of PEPC protein (Schüssler et al., 2016). Only few amino acid changes in exon 9 of PEPC1 seem to play a major role in altering the efficiency of the enzyme toward its function in the C_4_ pathway (Paulus et al., 2013). Inspection of the PEPC1 exon 9 amino acid sequence indicates that C_4_-typical changes, e.g., alanine to serine at position 780 (*Zea mays*; Bläsing et al., 2000) or 774 (*Flaveria*) and phenylalanine to valine at position 794 (*Z. mays*; Christin et al., 2007; Rosnow et al., 2014) are expectedly not present in the C_3_ and C_2_ Salsoleae species studied here, present in the two C_4_ species *H. scoparia* and *S. oppositifolia*, but are surprisingly absent in the C_4_ species *S. soda*. The entire amino acid sequence of PEPC1 exon 9 of *S. soda* is more similar to those of the C_3_ and C_2_ species than to the other studied Salsoleae C_4_ taxa. This indicates that either the PEPC efficiency in C_4_ function is improved by alternative amino acid changes (as described for Suaedoideae; Rosnow et al., 2015) or that the high expression of PEPC1 in the mesophyll cells is already sufficient for C_4_ function. PEPC1 has also been shown to be the C_4_-specific isoform in other C_4_ lineages of Caryophyllales, while e.g., in *Flaveria* (Asterales) a different PEPC isoform was recruited into the C_4_ cycle (Sunil et al., 2014).

Unexpectedly, the gene encoding the P-protein of the glycine cleavage system, GDC-P, which normally is involved in CO_2_ release in photorespiration, was expressed at a considerable amount not only in the C_3_ and C_2_ species but in all five Salsoleae species irrespective of the photosynthetic type. We therefore hypothesize that while most enzymes of the photorespiratory cycle should be absent from the mesophyll of C_4_ species because RuBisCO and the toxic products of its oxygenation reaction are lacking, others like GDC could still be present given their dual role also in C_1_ metabolism (Parys and Jastrzebski, 2008; Schulze et al., 2016).

Based on gene expression data, Lauterbach et al. (2016) speculated that the proteins encoded by PHT4.1 and PHT4.4 might be involved in the C_4_ syndrome, at least in *S. soda*. PHT4.1 and PHT4.4, which may function as ascorbate transporters, could play a role in protection against reactive oxygen species stress resulting from photostress in the mesophyll cells of NADP-ME C_4_ species by providing cells with the antioxidant ascorbate (Miyaji et al., 2015; Lauterbach et al., 2016). Consistent with the high expression level of PHT4.1 and PHT4.4 in the C_4_ species *S. soda* (Lauterbach et al., 2016), a significant increase of transcript abundance of these two genes was also observed in the two other C_4_ species, *H. scoparia* and *S. oppositifolia* (current study). Intriguingly, PHT4.1 transcripts were highly and PHT4.4 transcripts slightly more abundant in C_2_ *S. divaricata*, too. Thus, the results of the current study support a proposed function of the two PHT4 family proteins related to both C_4_ and C_2_ photosynthesis.

Asparagine synthetase, ASN, was found to be highly expressed in C_4_ leaves of *S. soda* when compared to C_3_ cotyledons of the same species (Lauterbach et al., 2016). ASN functions in ammonium metabolism, and asparagine is a key compound for nitrogen transport (Lam et al., 1996). Therefore, a possible functional connection between nitrogen metabolism and the switch from C_3_ to C_4_ was postulated (Lauterbach et al., 2016). In the current study, all C_4_ species showed high levels of transcripts encoding ASN, nevertheless transcript abundance in *S. soda* was much higher compared to the other species. In *Flaveria*, however, no significant differences of ASN expression between C_3_ *F. pringlei* and *F. robusta*, C_2_ *F. ramosissima*, and C_4_ *F. bidentis* and *F. trinervia* could be detected (see Supplementary Dataset 1, Gowik et al., 2011). In Cleomaceae on the other hand, ASN was also significantly upregulated in C_4_ *Gynandropsis gynandra* from leaf stage 3 onwards (i.e., leaves of an age 6 days onwards) compared to closely related C_3_ *Tarenaya hassleriana* (see Supplementary Datasets, Külahoglu et al., 2014). Thus, further investigation of up-regulation of ASN transcription in C_4_ species of Salsoleae might be worthwhile.

The PCA showed *S. divaricata* Pop-198 somewhat closer to the C_4_ species than *S. divaricata* Pop-184. Assuming that C_2_ species are true evolutionary intermediates with a gradual increase of C_4_-ness from C_3_ to C_4_ (Sage et al., 2012; Bräutigam and Gowik, 2016), gene expression profiles of the two populations of *S. divaricata* could imply that *S. divaricata* Pop-198 is closer to C_4_ than Pop-184. However, only two populations were studied and the variation of transcript abundance might as well reflect a generally higher plasticity of photosynthetic gene expression in C_2_ species. In a *Flaveria* study, a higher expression of NADP-ME was already present in more C_3_-like intermediate species, which linearly increased in the more advanced intermediates and peaked in the C_4_ species (Mallmann et al., 2014). This may be taken to indicate a more advanced C_4_ cycle in Pop-198 than in Pop-184, because NADP-ME is significantly more abundant in Pop-198. Nevertheless, a wide range of C_4_ genes, for example the first enzyme of the C_4_ cycle, PEPC, were highly expressed in both populations, implying a low-level but detectable C_4_ cycle in *S. divaricata* in general. However, a C_4_ cycle should result in less negative carbon isotope values; a result that was not observed in the current study. Transcript abundance of most of the core enzymes of photorespiration were highly increased in *S. divaricata* compared to both C_3_ and C_4_ species, which is consistent with expression patterns in C_2_ species of *Flaveria* (Gowik et al., 2011; Mallmann et al., 2014) and confirms the successful establishment of the C_2_ cycle in *S. divaricata*. These gene expression results thus complement anatomical findings, results of *in situ* immuno-localization with an antibody against GDC-P, and results of gas exchange measurements of *S. divaricata*, that all together identified *S. divaricata* as C_2_ species (Voznesenskaya et al., 2013).

C_4_ photosynthesis has been subdivided into three subtypes based on the main acting decarboxylases: NAD-ME, NADP-ME and PEP-CK. Recently, it was suggested to consider the PEP-CK type as a supplementary pathway to either NAD-ME or NADP-ME (Wang et al., 2014) because PEP-CK-only decarboxylation could not be observed. In subfamily Salsoloideae, the NAD-ME subtype is known from C_4_ species of the Caroxyloneae, and the NADP-ME subtype is known from C_4_ species of the Salsoleae (Akhani et al., 2007), whereas PEP-CK activity related to C_4_ photosynthesis has so far not been reported in this group. Interestingly, while according to their transcript patterns all of the three studied Salsoleae C_4_ species exclusively use NADP-ME as decarboxylase, we found PEP-CK abundant in both populations of *S. divaricata*. In *Flaveria*, PEP-CK was lowly expressed in C_2_ species and no differences in gene expression between the different photosynthetic types could be found (Gowik et al., 2011; Mallmann et al., 2014). In two C_2_ species of *Moricandia*, however, transcripts encoding PEP-CK showed enhanced abundance compared to C_3_ *Moricandia moricandioides* (Schlüter et al., 2016a). Also, Ala-AT transcripts were not increased in C_2_ *Moricandia* and NADP-ME transcripts only slightly enhanced (Schlüter et al., 2016a). Likewise, we observed that Ala-AT transcript abundance was not higher in *S. divaricata* and augmented transcript abundance of the decarboxylase NADP-ME was only observed in one population (Pop-198) when compared to *S. webbii*. It is tempting to speculate on a possible function of high PEP-CK abundance in a low level C_4_ cycle in the C_2_ species *Moricandia arvensis, M. suffruticosa*, and *S. divaricata*. One would expect, however, that genes encoding other proteins involved in the PEP-CK type C_4_ cycle, such as Ala-AT and Asp-AT, were also highly abundant, which at least in case of *S. divaricata* was not observed. High abundance of PEP-CK is inconsistent with a study investigating the decarboxylation enzymes of several plant groups, including few species of Salsoleae (Koteyeva et al., 2015). In this study, only low PEP-CK activity and PEP-CK protein abundance (using immunostaining) were found in *S. divaricata* (Koteyeva et al., 2015). These contrasting results could have different reasons. First, neither of the three anti-PEP-CK antibodies (anti-*Megathyrsus maximum*, anti-*Oryza sativa*, anti-*Ananas comosus*; Koteyeva et al., 2015) was able to bind to the PEP-CK gene that was upregulated in the current study. Second, post-transcriptional or post-translational regulation of PEP-CK could result in low abundance of PEP-CK protein. Third, variation in PEP-CK transcript abundance among populations of *S. divaricata* could occur. The latter explanation is at least partly supported because in the two studied populations transcript abundance of PEP-CK differed three-fold. Also, plasticity in the decarboxylation biochemistry of a supplemental PEP-CK pathway has been hypothesized (Furbank, 2011) and predicted in a modeling analysis (Wang et al., 2014) as well as observed in the C_4_ species maize (Pick et al., 2011) and C_4_ *G. gynandra* (Sommer et al., 2012). Information about the localization of PEP-CK within the leaf could clarify a possible function of PEP-CK in *S. divaricata*, and also in C_3_-C_4_ *Moricandia* species.

## Conclusion

The overall gene expression pattern, as visualized by PCA, showed photosynthetic type as the main factor separating the different Salsoleae species. C_2_ type *S. divaricata* was intermediate between C_3_ *S. webbii* and the three C_4_ species. Despite major differences in life cycle, habit and photosynthesis during seedling stage, the C_4_ species showed similar gene expression profiles of C_4_ genes and photorespiratory genes. Most C_4_ genes were highly abundant in all three C_4_ species. Further, our results suggested that PEPC1 was the C_4_-specific isoform in Salsoleae, as found for other C_4_ lineages of Caryophyllales. The protein, however, lacks C_4_ typical amino acid changes in *S. soda*. Two recently proposed transporters from the PHT4 protein family might not only be involved in C_4_ photosynthesis, but also be active in C_2_ photosynthesis in Salsoleae. Here, they might be involved in protection against reactive oxygen species resulting from the absence of most photorespiratory reactions in the mesophyll. The transcript profile of the C_2_ species *S. divaricata* was mostly comparable with that observed in C_2_ species of *Flaveria* or *Moricandia*. Moreover, in one population of *S. divaricata*, Pop-198, the transcript pattern of C_4_ genes implied a slightly more advanced C_4_ cycle than in Pop-184. However, a C_4_ cycle is not detectable in the carbon isotope values, and this species is not functioning as a C_4_ plant physiologically (Voznesenskaya et al., 2013). Also, a function of PEP-CK in C_2_ photosynthesis in *S. divaricata* is likely, because PEP-CK was highly increased compared to C_3_ and C_4_ species of Salsoleae, a result that was also observed in C_2_ *Moricandia*, but needs further investigations.

## Author contributions

ML designed the research, performed experiments, analyzed and interpreted the data, and wrote the manuscript. HS analyzed and interpreted the data, and critically revised the manuscript. KB performed experiments. TH designed the research and critically revised the manuscript. PW designed the research and critically revised the manuscript. UG designed the research, analyzed and interpreted the data, and critically revised the manuscript. GK conceived and designed the research, interpreted the data, and critically revised the manuscript.

### Conflict of interest statement

The authors declare that the research was conducted in the absence of any commercial or financial relationships that could be construed as a potential conflict of interest.

## Supplementary material

Supplementary Table S1 Details of *de novo* transcriptome assemblies using SOAPdenovo-Trans and Trinity.

Supplementary Table S2 Mapping of reads, transcript abundance and statistical tests of differentially gene expression using edgeR. Key C4 genes and core enzymes of photorespiration are annotated in column B and C. Hs, *Hammada scoparia*; Sd184, *S. divaricata* Pop-184; Sd198, *S. divaricata* Pop-198; So, *S. oppositifolia*; Ss, *S. soda*; Sw, *S. webbii*.
