# Supplementary material for: De novo Transcriptome Assembly and Comparison of C3, C3-C4, and C4 Species of Tribe Salsoleae (Chenopodiaceae)
Source: Front Plant Sci. 2017 Nov 14;8:1939. doi: 10.3389/fpls.2017.01939 (PMC5694442; doi:10.3389/fpls.2017.01939)
Supplement: Supplementary file 3 [file Image1.PDF]

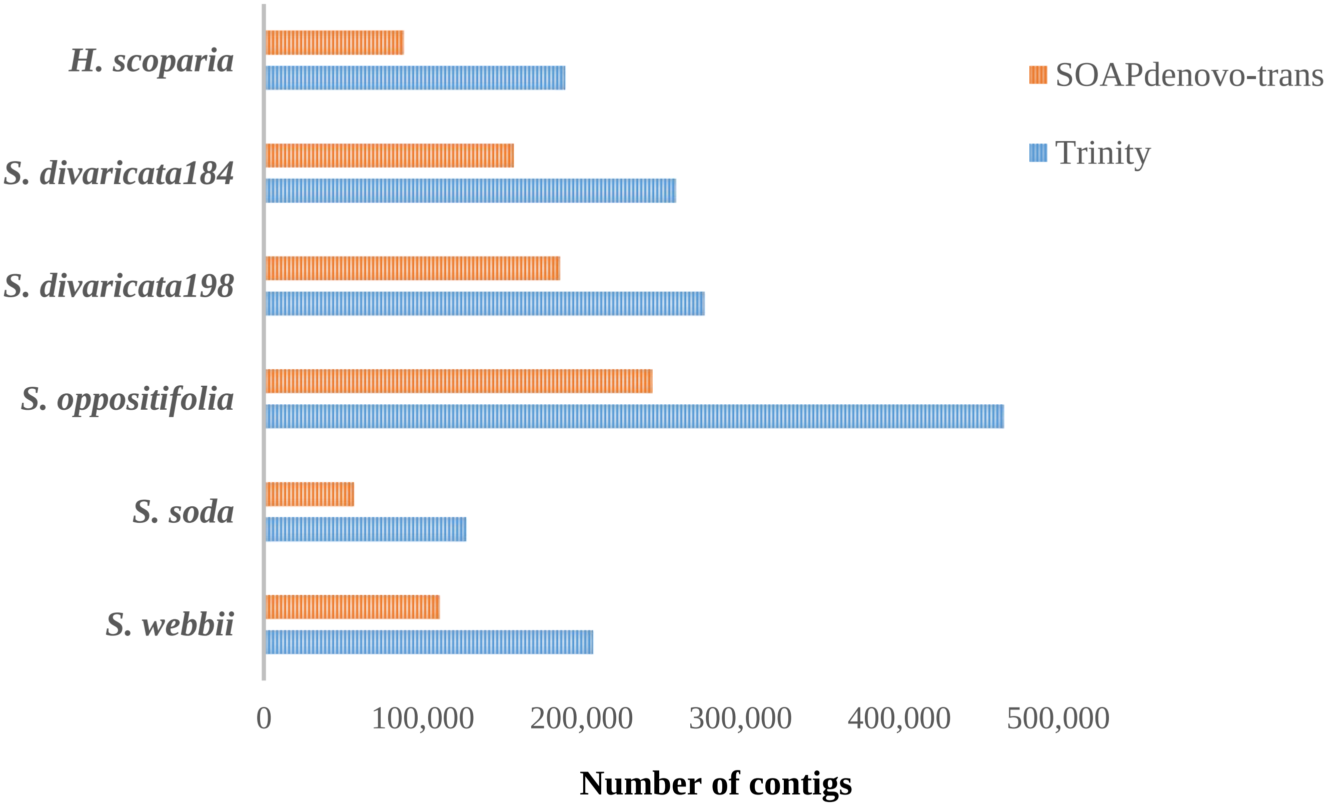

Supplementary Figure S1. Number of contigs in all six assemblies using the two different assemblers SOAPdenovo-Trans and Trinity.
