# Supplementary material for: De novo Transcriptome Assembly and Comparison of C3, C3-C4, and C4 Species of Tribe Salsoleae (Chenopodiaceae)
Source: Front Plant Sci. 2017 Nov 14;8:1939. doi: 10.3389/fpls.2017.01939 (PMC5694442; doi:10.3389/fpls.2017.01939)
Supplement: Supplementary file 4 [file Image2.PDF]

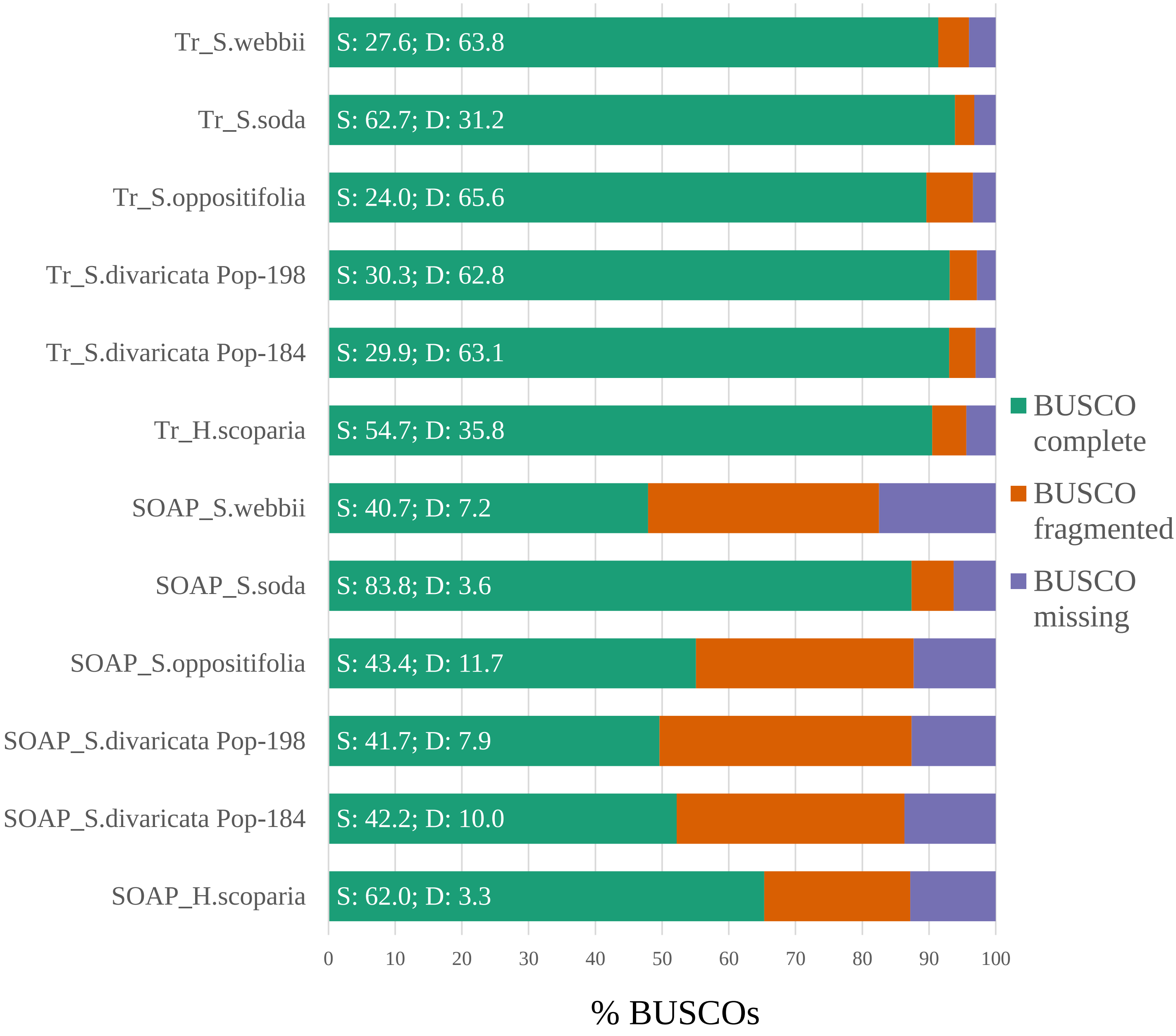

Supplementary Figure S2. Quality assessment of transcriptome *de novo* assemblies of the two assemblers SOAPdenovo-Trans (SOAP) and Trinity (Tr) using the plant data set in BUSCO. BUSCOs are categorized into complete (green), fragmented (orange), and missing (purple). Numbers within category ‘complete’ indicate the subcategories ‘complete and single-copy’ (S) and ‘complete and duplicated’ (D).
