# Supplementary material for: De novo Transcriptome Assembly and Comparison of C3, C3-C4, and C4 Species of Tribe Salsoleae (Chenopodiaceae)
Source: Front Plant Sci. 2017 Nov 14;8:1939. doi: 10.3389/fpls.2017.01939 (PMC5694442; doi:10.3389/fpls.2017.01939)
Supplement: Supplementary file 5 [file Image3.PDF]

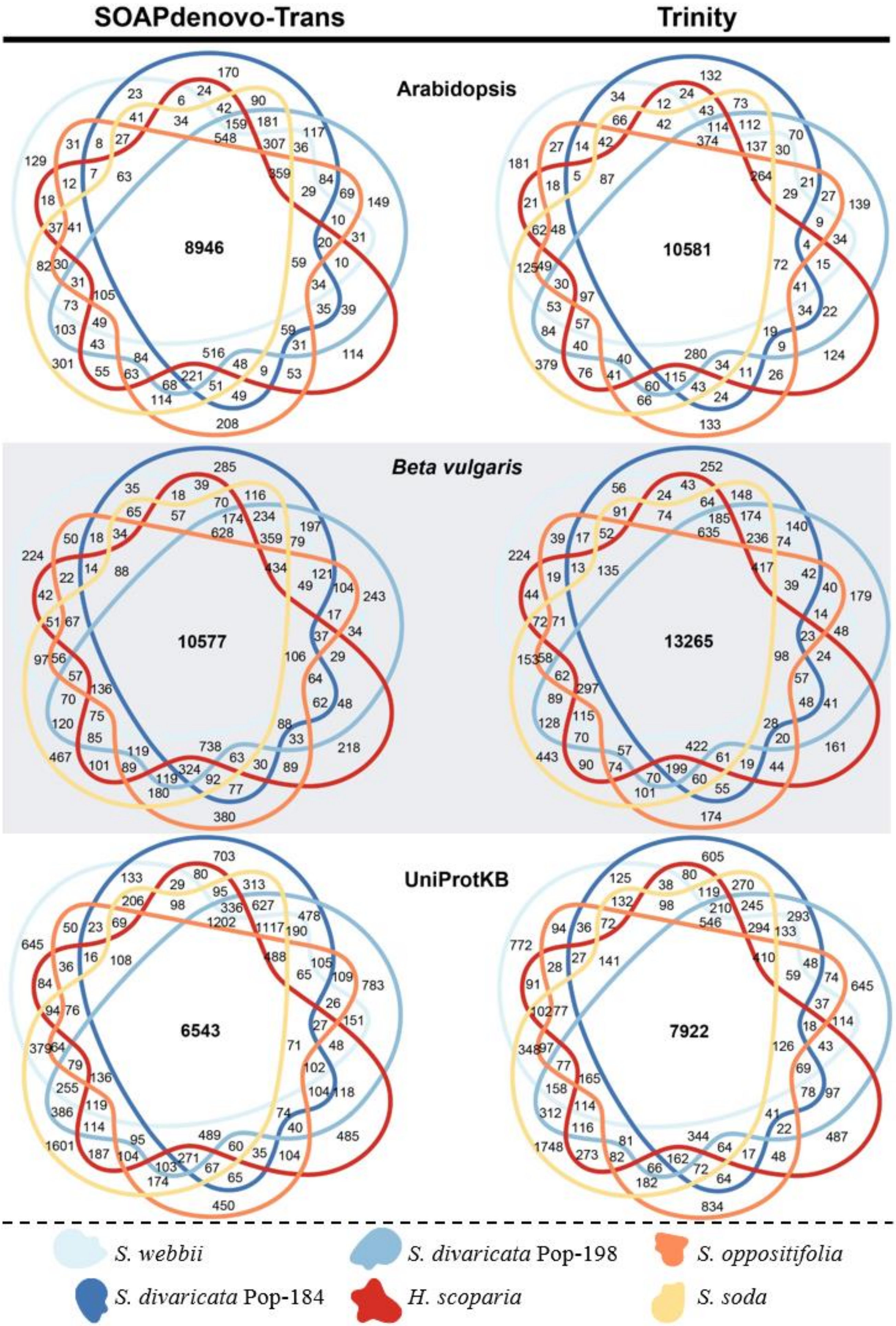

Supplementary Figure S3. Shared contigs between the six *de novo* assemblies (SOAPdenovo-Trans or Trinity for assembly) using three different references Arabidopsis, *Beta vulgaris*, and UniProtKB for annotation of contigs visualized as Venn diagrams.
