# Supplementary figures and images for: De novo Transcriptome Assembly and Comparison of C3, C3-C4, and C4 Species of Tribe Salsoleae (Chenopodiaceae)
Source: Front Plant Sci. 2017 Nov 14;8:1939. doi: 10.3389/fpls.2017.01939 (PMC5694442; doi:10.3389/fpls.2017.01939)

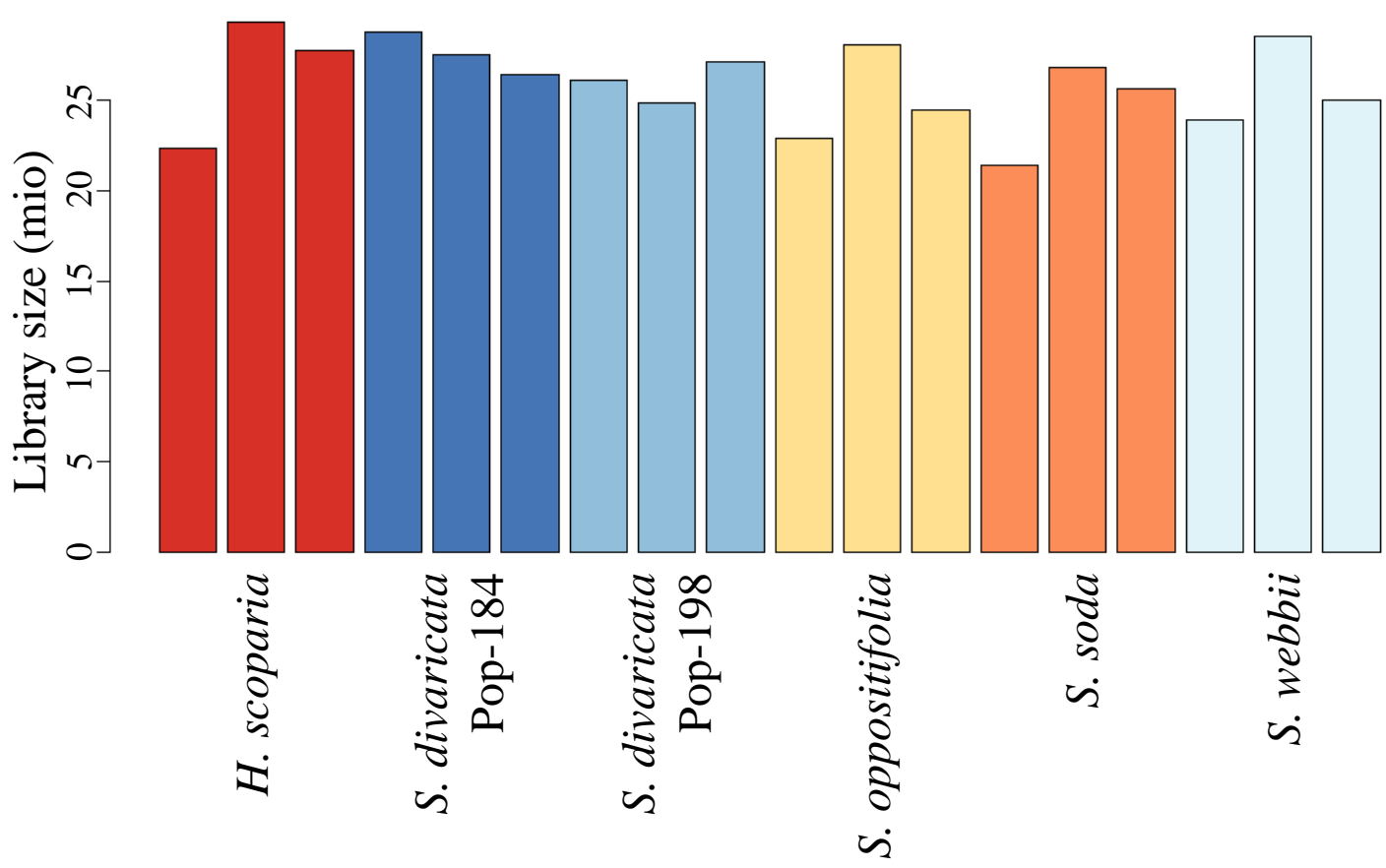

**Supplementary Figure S4.** Total read counts per sample of all replicates.

Supplement: Supplementary file 6 [file Image4.PDF]
