# Supplementary material for: De novo Transcriptome Assembly and Comparison of C3, C3-C4, and C4 Species of Tribe Salsoleae (Chenopodiaceae)
Source: Front Plant Sci. 2017 Nov 14;8:1939. doi: 10.3389/fpls.2017.01939 (PMC5694442; doi:10.3389/fpls.2017.01939)

Supplementary Figure S5. Principal component analysis showing the first three components, which together explain 76.32% of the variation. **A**) first (X axis) and second (Y axis) components; **B**) second (X axis) and third (Y axis) components; **C**) first (X axis) and third (Y axis) components.

A

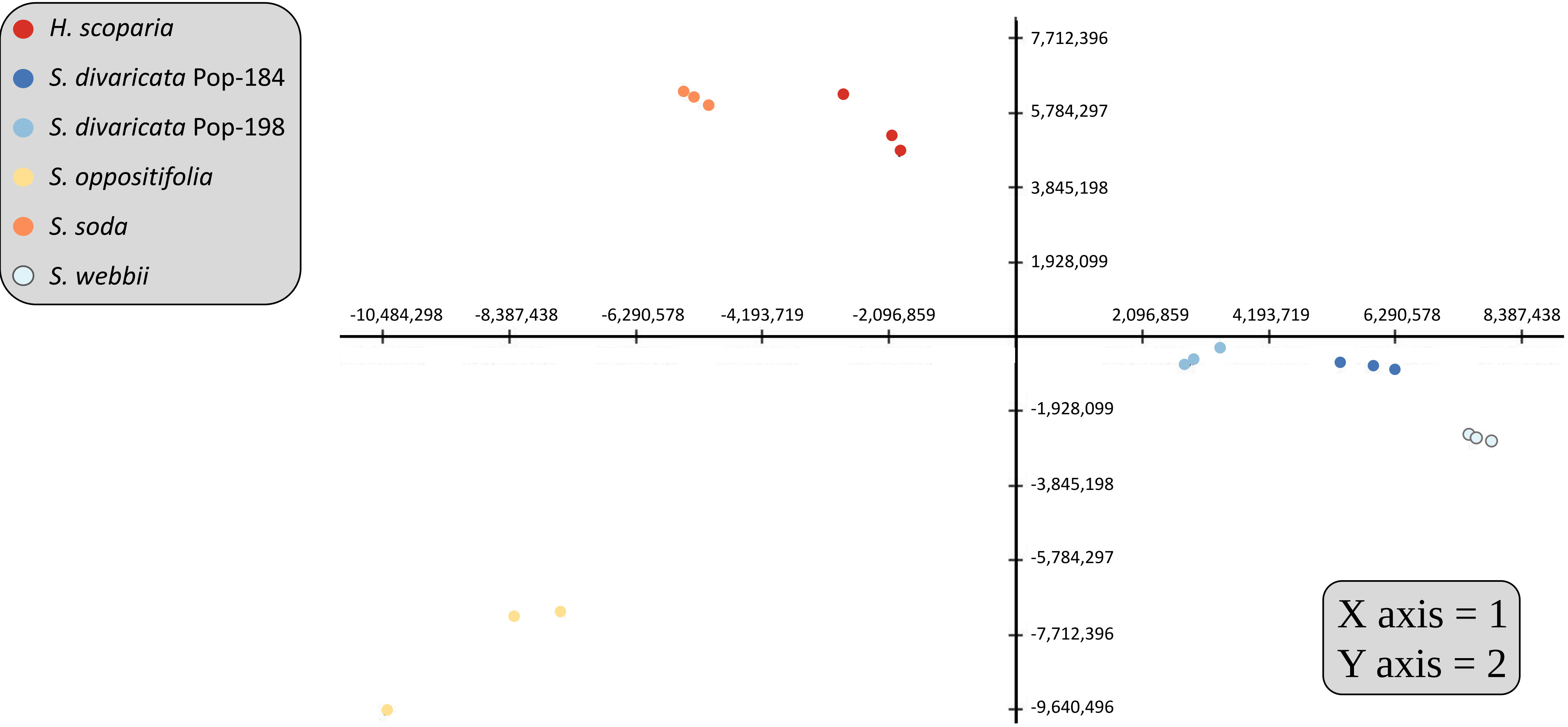

Supplementary Figure S5. Principal component analysis showing the first three components, which together explain 76.32% of the variation. **A**) first (X axis) and second (Y axis) components; **B**) second (X axis) and third (Y axis) components; **C**) first (X axis) and third (Y axis) components.

B

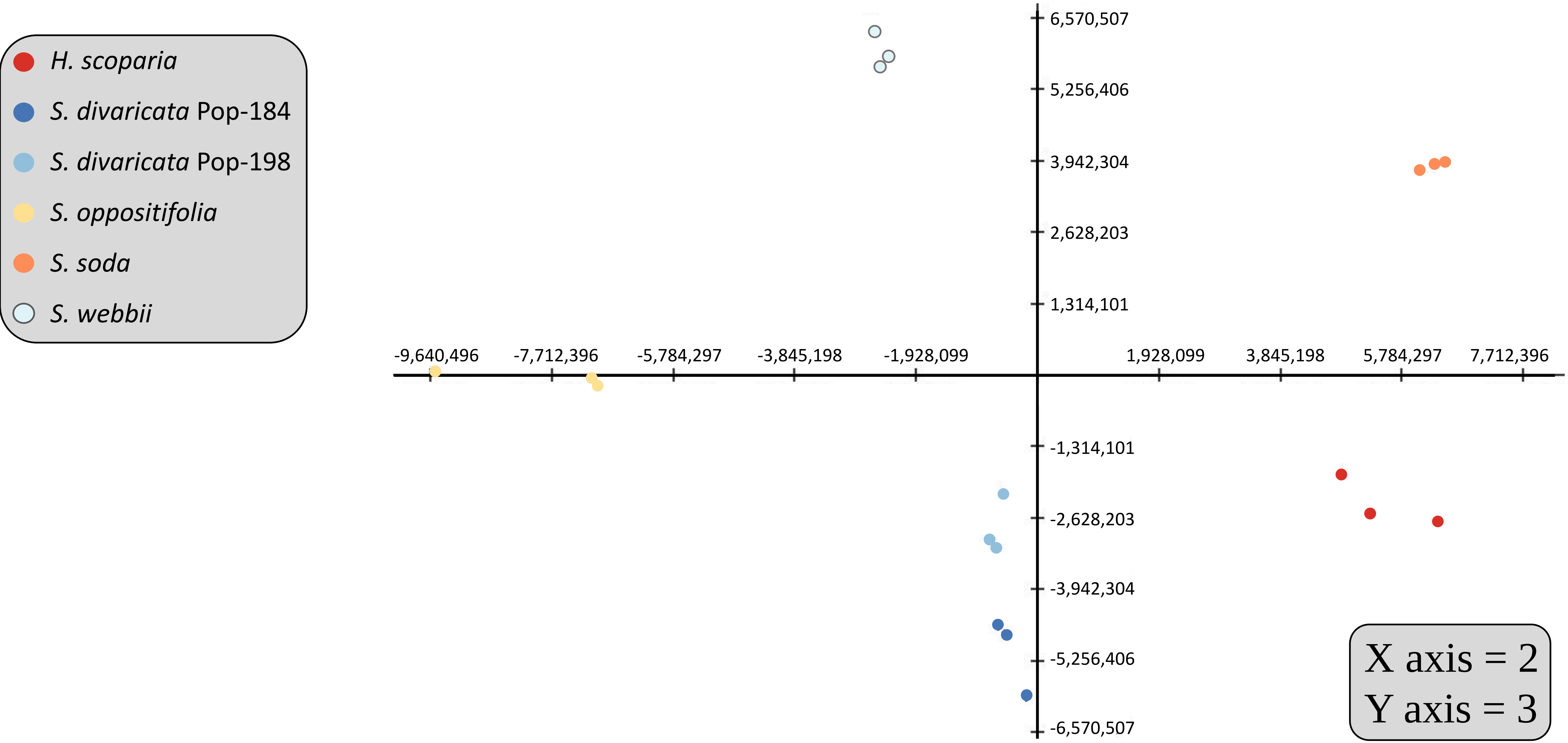

Supplementary Figure S5. Principal component analysis showing the first three components, which together explain 76.32% of the variation. **A**) first (X axis) and second (Y axis) components; **B**) second (X axis) and third (Y axis) components; **C**) first (X axis) and third (Y axis) components.

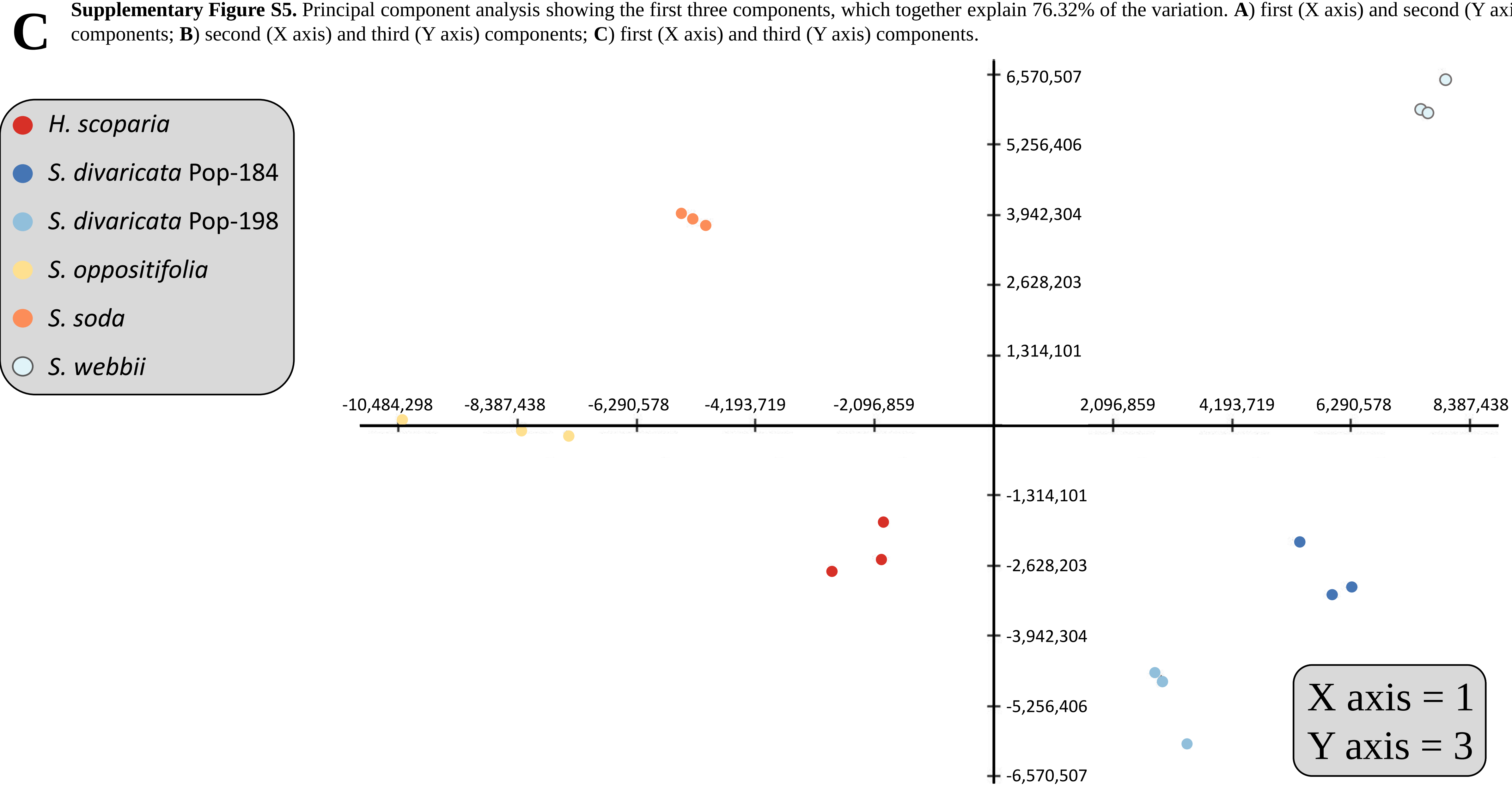

Supplement: Supplementary file 7 [file Image5.PDF]
